# Pregabalin inhibits *in vivo* and *in vitro* cytokine secretion and attenuates spleen inflammation in Lipopolysaccharide/Concanavalin A -induced murine models of inflammation

**DOI:** 10.1038/s41598-020-61006-1

**Published:** 2020-03-04

**Authors:** Eman Y. Abu-rish, Ahmad T. Mansour, Hebah T. Mansour, Lina A. Dahabiyeh, Shereen M. Aleidi, Yasser Bustanji

**Affiliations:** 1 Department of Biopharmaceutics and Clinical Pharmacy, School of Pharmacy, The University of Jordan, Amman, 11942 Jordan; 2 Department of Pathology and Microbiology and Forensic Medicine, School of Medicine, The University of Jordan, Amman, 11942 Jordan; 3 Department of Pharmaceutical Sciences, School of Pharmacy, The University of Jordan, Amman, 11942 Jordan; 4 Hamdi Mango Centre for Scientific Research, The University of Jordan, Amman, Jordan

**Keywords:** Acute inflammation, Neuroimmunology, Neuropathic pain, Epilepsy

## Abstract

Immune system alteration has been implicated in the pathogenesis of chronic pain conditions, epilepsy and generalized anxiety disorder. Targeting cytokines has recently been proposed for the management of such conditions. Pregabalin (PGB) is an antiepileptic agent used for the management of these conditions. However, little is known about its immunomodulatory effects on cytokine secretion *in vivo* and *in vitro*. Hence, a mitogen (Lipopolysaccharide [LPS] or Concanavalin A [ConA])-induced murine model of inflammation was used to investigate the effect of PGB on *in vivo* and *in vitro* IL-1β, IL-6, TNF-α and IL-2 cytokine secretion using ELISA. In addition, PGB effect on spleen histology, as a lymphoid organ, was examined. Our results revealed that PGB significantly inhibited the secretion of ConA-induced IL-6 secretion, basal and ConA-induced TNF-α and IL-2 secretion in splenocytes *in vitro*. *In vivo*, PGB inhibited basal and LPS/ConA-induced IL-6 and TNF-α secretion in addition to LPS-induced IL-1β and ConA-induced IL-2 secretion. Moreover, PGB attenuated mitogen-induced inflammatory changes in the spleen. These findings provide an evidence of the anti-inflammatory properties of PGB on cytokine secretion and lymphoid organ inflammation. This might give insights into the role of PGB in the management of the inflammatory state in PGB-indicated conditions.

## Introduction

Several antiepileptic drugs have documented immunomodulatory effects, in particular, the old generation agents^1–5^. Pregabalin (PGB), is one of the new generation antiepileptic agents that was approved by the U.S Food and Drug Administration (FDA) in 2004^6^. It acts through binding to a specific subunit in the voltage-dependent calcium channels in the brain^6^. It is indicated for the treatment of several neuropathic pain conditions, fibromyalgia, and generalized anxiety disorder, and it is also indicated as an adjunctive therapy for the management partial onset seizure^6^. The involvement of inflammatory mechanisms in the pathogenesis of these conditions has increasingly been reported. Neuroinflammation and peripheral inflammation is involved in chronic pain conditions where elevated levels of local and serum IL-6, TNF-α, IL-1β and IL-2 have been documented^7,8^. Systemic IL-6, TNF-α, IL-1RA and IL-2 were also elevated in patients with fibromyalgia^9,10^. Furthermore, a large panel of chemokines were elevated in the plasma and in the cerebrospinal fluid (CSF) in patients with this condition^11^. Similarly, elevated levels of the proinflammatory cytokines IL-6, TNF-α, IL-1β and IL-2 have been reported in epilepsy^12–16^. In patients with generalized anxiety disorders, an elevated level of several proinflammatory cytokines, including IL-6, TNF-α, IL-1β and IL-2 have been reported^17^. Thus, peripheral inflammation is observed in the principal PGB-indicated conditions which share the alteration in IL-6, TNF-α, IL-2 and IL-1β cytokines level. Yet, reports on PGB immunomodulatory and anti-inflammatory effects are a few and focused mainly on *in vitro* assays, local inflammatory changes within the nervous system, and attenuation of the secretion of only IL-1β and TNF-α cytokines^18–20^. In addition, PGB effects on cytokines secretion in immune cells, such as isolated splenocytes and peritoneal macrophages (PMs), and its effect on lymphoid organs, were not examined before. Therefore, there is a need to perform a simultaneous assessment of the effect of PGB on the secretion of the cytokines that were commonly elevated in the aforementioned PGB-indicated conditions (IL-6, TNF-α, IL-1β and IL-2) and to expand the investigation to its effect on lymphoid organs and cytokine secretion in immune cells.

As peripheral inflammation and elevated systemic cytokines levels were demonstrated in the above-mentioned PGB-indicated conditions, we investigated in this study the effect of PGB on murine models of peripheral inflammation. In this study, we used for the first time, LPS and ConA-induced murine models of inflammation to examine the effect of PGB on peripheral proinflammatory cytokine secretion (IL-6, TNF-α, IL-1β and IL-2) *in vitro* and *in vivo* in BALB/c mice. LPS-model of inflammation has been employed before to study neuroinflammatory conditions, seizure and anxiety disorders in mice^21–25^, while ConA was used to investigate T-cell function in patients with fibromyalgia^26^. Additionally, in this study, the effect of PGB on mitogen-induced inflammatory changes in the spleen, as a lymphoid organ, was also examined for the first time. Regarding *in vitro* investigation, the lack of reports that examined the effect of PGB on the secretion of cytokines in immune cells prompted us to investigate such effects of PGB on basal and mitogen-induced proinflammatory cytokines secretion in splenocytes and peritoneal macrophages (PMs).

## Methods

### Animals

Female BALB/c mice (6 to 8 weeks old; 18–22 g weight) were provided by the animal house at the University of Jordan, Amman, Jordan. Animals were housed in plastic cages and were kept under the standard laboratory conditions (temperature, 21–23 °C, humidity, 55–60% and 12 h light/dark cycle) with free access to laboratory diet and water. Ethical approval of the study protocol was obtained by the scientific committee at the School of Pharmacy, The University of Jordan. All methods were carried out in accordance with the relevant guidelines and regulations.

### Reagents

PGB powder was kindly provided by Sana Pharma (Amman, Jordan). RPMI-1640 medium, high glucose DMEM media, fetal bovine serum (FBS), penicillin-streptomycin solution, L-glutamine, thiazoyl blue tetrazolium bromide (MTT), ConA (from *Canavalia ensiformis*, catalogue number L 7647) and LPS (from *Escherichia coli* O111:B4, catalogue number L 4130) were all purchased from Sigma-Aldrich Chemical Co. (USA). Mouse Ready-SET-Go ELISA kits for murine IL-6, TNF-α, IL-1β and IL-2 were obtained from ThermoFisher Scientific (USA). Eosine Y reagent and Haematoxylin reagent were brought from Gainland Chemical Company (UK) and Fluka Chemicals (Germany), respectively.

### PGB preparation

A stock solution of 10 mg/ml PGB was prepared in normal saline (NS) for *in vivo* experiments and in phosphate-buffered saline (PBS) for *in vitro* experiments.

### Dosage and treatment protocol for *in vivo* studies

**Figure 1:**
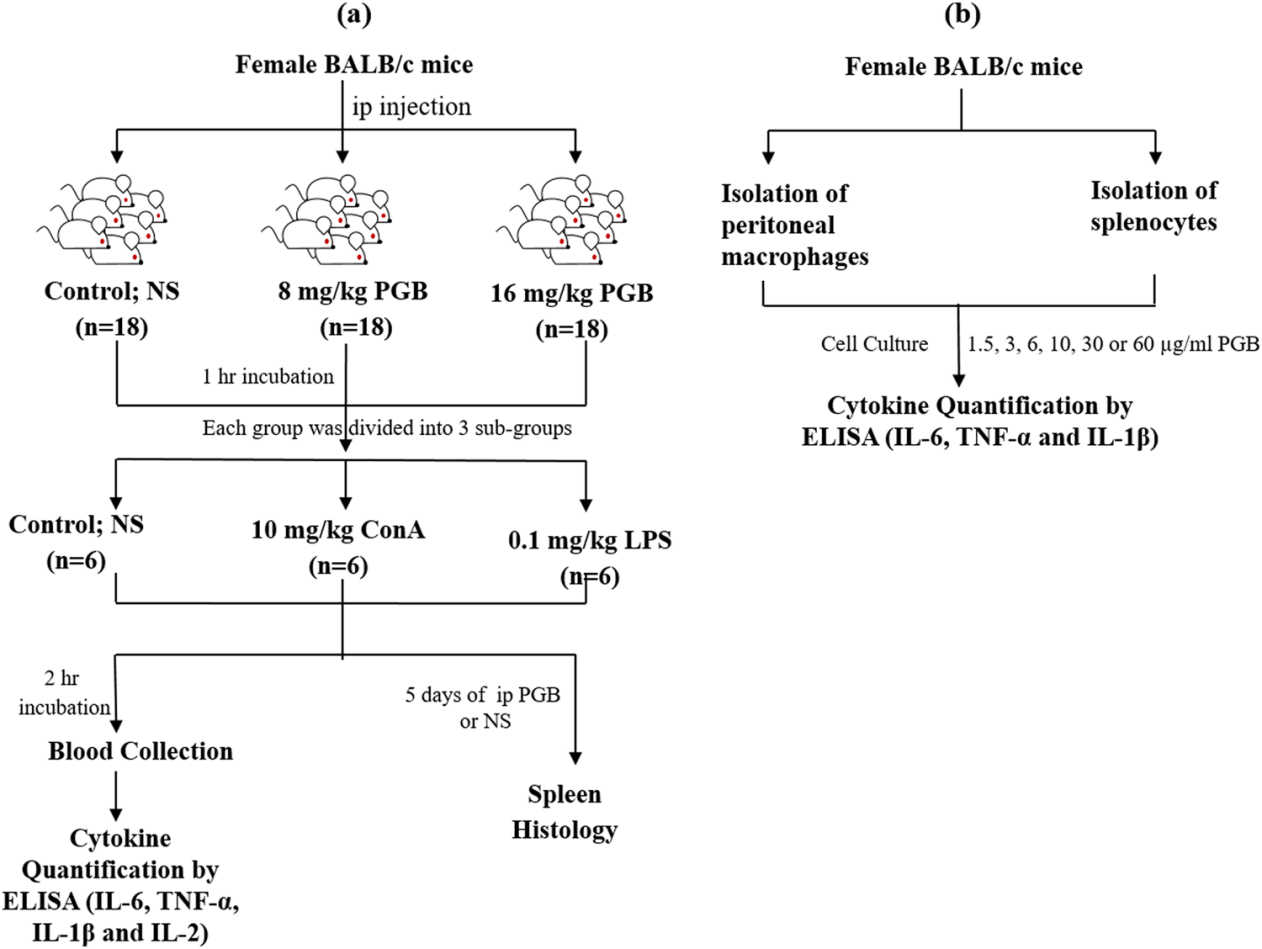
Work flow for (**a**) *in vivo* and (**b**) *in vitro* investigations. NS, normal saline; PGB, pregabalin; ConA, concanavalin A; LPS, lipopolysaccharide.

Three groups of mice (n = 18) were either treated with NS intraperitoneally (i.p) (control group) or with 8 or 16 mg/kg of PGB (i.p), corresponding to 1- and 2-times the maximum human therapeutic dose, respectively (Fig. 1a). Mice were incubated for 1 hour with NS or PGB before dividing each group into 3 sub-groups (n = 6), which were either treated with 0.1 mg/kg of LPS (i.p) or 10 mg/kg of ConA (i.p) or were left without additional treatment. For serum cytokines quantification, mice were then incubated for additional 2 hours in the presence or absence of the mitogen before being anesthetized with diethyl ether inhalation prior to blood collection. For the histological examination of the spleen, mice in the treatment groups continued to receive a daily injection of PGB - at the indicated doses- or NS for additional 5 days before being anesthetized with diethyl ether inhalation and scarified for spleen extraction.

### Serum preparation for cytokines quantifications

Blood was collected from retro-orbital plexus into silica gel-containing plain tubes. Serum was prepared by leaving the blood to clot at room temperature (RT) for 30 minutes followed by centrifugation at 3000 rpm for 10 minutes at 4 °C. Sera were frozen at −80 °C immediately after collection. ELISA measurement of IL-6, TNF-α, IL-1β and IL-2 levels was performed as per manufacturer’s protocols (ThermoFisher Scientific, USA). Ranges of detection were 4–500; 8–1000; 8–1000; 2–200 pg/mL, respectively.

### Histological examination of the spleen

Collected spleens were immediately fixed in formalin. Sections of 5-µm thickness were prepared using microtome and stained with Hematoxylin & Eosin (H&E). Histologic changes were examined by a blinded pathologist using Olympus light microscope.

### Preparation of Single splenocyte cell suspension (SSCS)

Spleens were collected in RPMI-1640 medium supplemented with 10% FBS under aseptic conditions. Spleens were minced between the frosted edges of two microscopic slides and the cells were then pooled and centrifuged at 1200 rpm for 7 min at 4 °C. The red blood cells (RBC) within the pellets were then lysed at RT for 3 min using RBC lysis buffer as previously described^27,28^. Splenocytes were then suspended in 1 mL of RPMI-10% FBS and viable splenocyte count was determined using trypan blue dye exclusion method^29^.

### Isolation of peritoneal macrophages (PMs)

Peritoneal cells were collected under aseptic conditions through three-time peritoneal lavage, each with fresh 5 ml of cold PBS. Cells were pooled, washed with cold PBS and then suspended in complete medium and plated for two hours for PMs to adhere. Non-adherent cells were washed twice with PBS^30^.

### Cell cultures

Two million cell/ ml of splenocytes or peritoneal cells were cultured in complete RPMI-1640 medium or complete high-glucose DMEM medium, respectively. Both media were supplemented with 10% FBS, 2 mM L-glutamine, 100 U/ml penicillin and 100 μg/ml streptomycin. Cultures were maintained at 5% CO_2_/ 37 °C. For cell proliferation assay and *in vitro* cytokine secretion assessment, splenocytes were treated immediately while peritoneal cells were left for 2 hours to allow the adherence of PMs. Cultures of both cell types were pre-treated for 1 hour with PGB at a final concentration of 1.5, 3, 6, 10, 30 or 60 µg/ml, or were left untreated (Fig. 1b). Splenocytes were then co-treated with 1 µg/ml ConA while PMs were co-treated with 100 ng/ml LPS, or both cultures were left without further treatment, for 24, 48 or 72 hours. PGB (3–30 µg/ml) concentration range was chosen according to previous reports in mice and on therapeutic concentrations in human^20,31^. Concentrations lower and higher than the previously reported concentrations were also tested (1.5 µg/ml; 60 µg/ml, respectively).

### *In vitro* cell proliferation assay

In flat-bottomed 96-well microplates, 4 × 10^5^ cells/200 µl/well of splenocytes or peritoneal cells were cultured as described above. Two hours prior to the end of each incubation interval, MTT assay was performed^32^. Optical density (OD) was measured at 570 nm using a microplate reader (Biotek, USA).

### Assessment of cytokine secretion *in vitro*

Splenocytes or PMs were cultured in 24-well plates and treated as described under “Cell culture” above. At the end of each incubation interval, culture supernatants were collected and immediately frozen at −80 °C. Levels of IL-6, TNF-α, IL-1β and IL-2 were measured by ELISA as per manufacturer’s protocols (ThermoFisher Scientific, USA).

### Statistical analysis

One-way ANOVA followed by Dunnett’s post-hoc analysis were used for data analysis using Prism 5 software (GraphPad, USA). All data are presented as means ± standard error of the means (SEM). *P*-values are indicated with asterisks as follows: *P < 0.05, **P < 0.01, ***P < 0.001.

## Results

### Effect of PGB on splenocytes and PMs cell viability in the presence or absence of mitogens (ConA or LPS)

PGB, at all concentrations; 1.5, 3, 10, 30, 60 (µg/ml) and over 24, 48 and 72 hours, had no statistically significant effect on the viability of both splenocytes and PMs, as compared to PGB-untreated control group neither in the presence nor in the absence of ConA/LPS (Supplementary Table S1).

### Effect of PGB on basal and mitogen-induced cytokine secretion *in vitro*

#### Effect on isolated splenocytes

**Figure 2:**
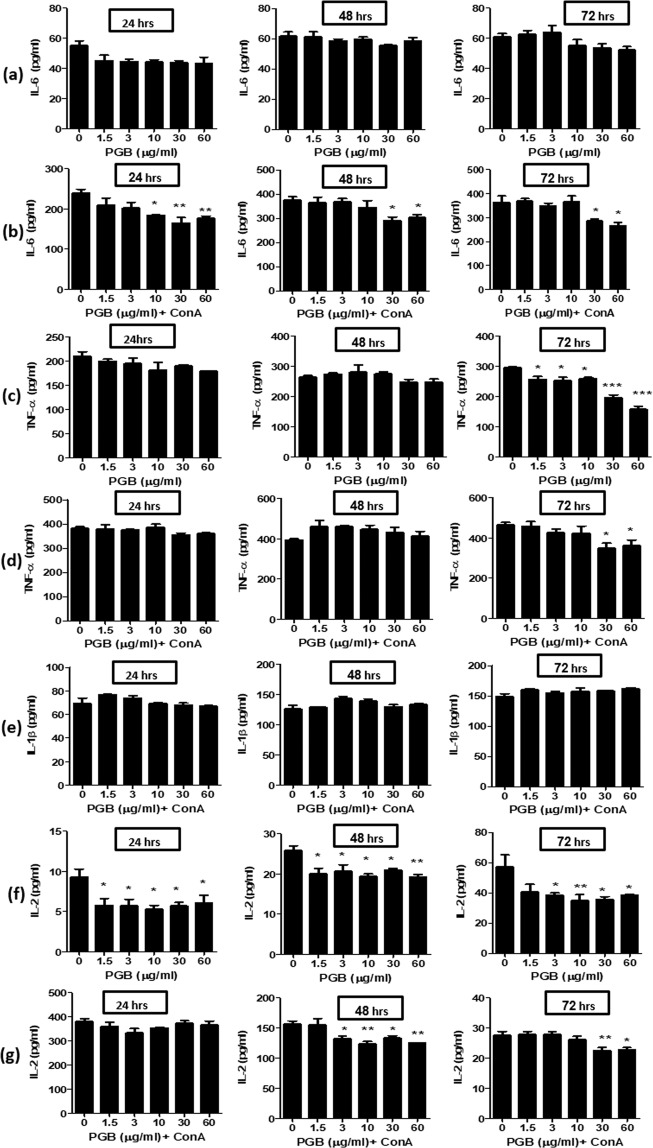
Effect of PGB on IL-6, TNF-α, IL-1β and IL-2 secretion in splenocytes. (**a**) basal IL-6 secretion, (**b**) ConA-induced IL-6 secretion, (**c**) basal TNF-α secretion, (**d**) ConA-induced TNF-α secretion, (**e**) ConA-induced IL-1β secretion, (**f**) basal IL-2 secretion, (**g**) ConA-induced IL-2 secretion. Splenocytes were treated with PGB alone (1.5, 3, 10, 30, 60 µg/ml) or were pre-treated with PGB for 1 hour and then co-treated with ConA (1 µg/ml). Cells were incubated for 24, 48 and 72 hours. ConA: concanavalin A, PGB, Pregabalin. (Data represent mean ± SEM; n = 3; ANOVA; Dunnett’s post-hoc analysis; *P < 0.05, **P < 0.01, ***P < 0.001).

The effect of PGB on cytokine secretion *in vitro* was first investigated in isolated splenocytes, in the presence or absence of ConA. As presented in Fig. 2(a,b), treatment of splenocytes with PGB resulted in a significant inhibition of ConA-induced IL-6 secretion at the concentrations 30–60 µg/ml, over the three time points, as compared to PGB-untreated control group (p = 0.0044; 0.0156; 0.0041 for 24, 48 and 72 hours, respectively). However, PGB had no statistically significant effect on basal IL-6 secretion.

Treatment of splenocytes with PGB, resulted in a significant inhibition of basal and ConA-induced TNF-α secretion after 72 hours of incubation only (p < 0.0001; p = 0.0232, respectively). Whilst the basal TNF-α secretion was inhibited across all the concentrations, the ConA-induced secretion was only inhibited at 30–60 µg/ml, as compared to PGB-untreated control group (Fig. 2c,d, respectively). PGB, across the whole concentration range, had no significant effect on ConA-induced IL-1β secretion at the three time points, as compared to PGB-untreated control group (Fig. 2e). The effect of PGB on basal IL-1β secretion could not be reliably determined as the levels were below the detection limit of the assay ( < 8 pg/ml, data not shown).

Basal IL-2 secretion in splenocytes was significantly inhibited with PGB across the whole concentration range and over the three time points (p = 0.0172; 0.0094; 0.0223, respectively), while ConA-induced IL-2 secretion was only inhibited at 48 (p = 0.0028) and 72 hours (p = 0.0058), at the concentration ranges 3–60 µg/ml and 30–60 µg/ml, respectively.

#### Effect on isolated PMs

Our results showed that PGB inhibited the secretion of a range of cytokines in ConA-treated or untreated splenocytes. Therefore, we next investigated the effect of PGB on LPS-induced cytokines secretion in PMs. Treatment of PMs with PGB had no effect on both basal and LPS-induced IL-6 and TNF-α secretion at the three time points (Supplementary Fig. S1a–d). IL-1β was undetectable at basal conditions and was only induced after treatment of PMs with LPS (100 ng/ml) for 72 hours where PGB had no effect on its secretion (Supplementary Fig. S1e). The effect of PGB on LPS-induced IL-2 was not examined as LPS does not induce IL-2 secretion^33^.

### Effect of PGB on basal and mitogen (ConA or LPS)-induced cytokine secretion *in vivo*

**Figure 3:**
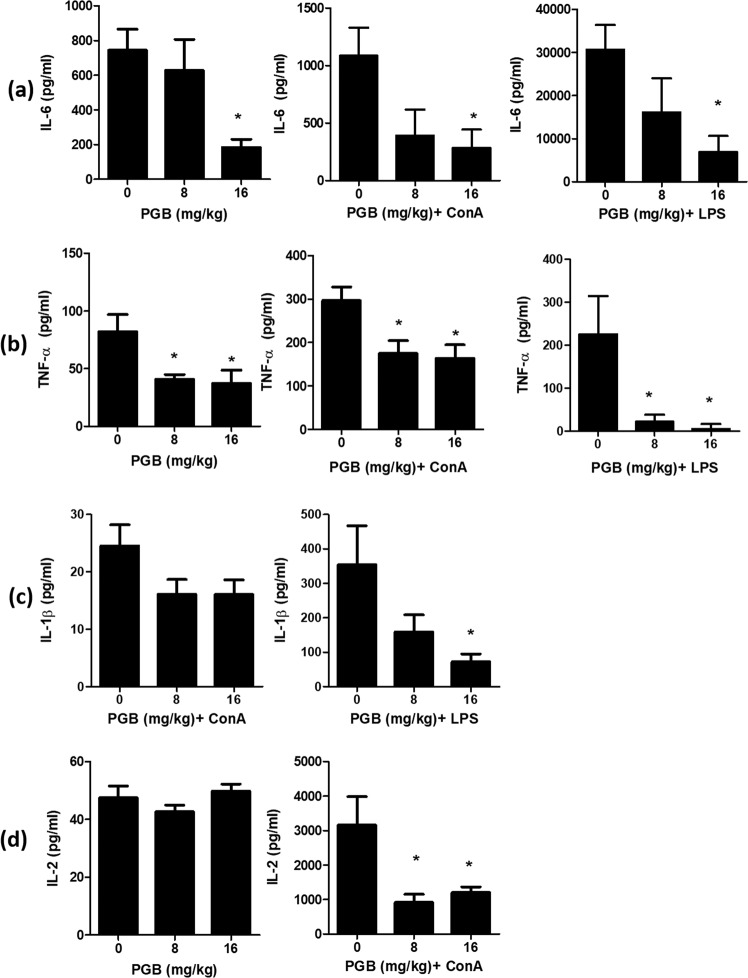
Effect of PGB on basal, ConA- and LPS-induced cytokine secretion *in vivo* (**a**) IL-6 secretion, (**b**) TNF-α secretion, (**c**) IL-1β secretion, and (**d**) IL-2 secretion. Mice were treated with PGB (0, 8, 16 mg/kg) alone for 3 hours or were pre-treated with PGB for 1 hour and then co-treated with ConA (10 mg/kg) or LPS (0.1 mg/kg) for another 2 hours. ConA: concanavalin A, LPS: lipopolysaccharide, PGB, pregabalin, (Data represent mean ± SEM; n = 6; ANOVA; Dunnett’s post-hoc analysis; *P < 0.05).

As PGB inhibited cytokine secretion in splenocytes *in vitro* with no effects on cytokines secretion in PMs, we next investigated whether it has similar effect on cytokines secretion *in vivo* in the presence or absence of ConA/LPS. PGB significantly inhibited basal, ConA- and LPS-induced IL-6 secretion at 16 mg/kg dose (p = 0.0199; 0.0489; 0.048, respectively) (Fig. 3a) while it inhibited basal, ConA- and LPS-induced TNF-α secretion at both doses tested; 8 and 16 mg/kg (p = 0.0217; 0.0249; 0.0232, respectively) (Fig. 3b). PGB significantly inhibited LPS-induced IL-1β secretion at 16 mg/kg (p = 0.0266), while it had no significant effect on ConA-induced IL-1β secretion under the experimental conditions used (Fig. 3c). IL-1β was undetectable at basal conditions, and therefore, the effect of PGB on IL-1β level could not be examined (<8 pg/ml, data not shown). With regards to IL-2 secretion, PGB had no effect on basal IL-2 secretion at 8 and 16 mg/kg doses while it significantly inhibited ConA-induced IL-2 secretion at the same doses (p = 0.0103) (Fig. 3d). All comparisons were made against PGB-untreated control group in the presence or absence of ConA/LPS.

### Effect of PGB on spleen histology in the presence or absence of mitogens (ConA or LPS)

As PGB inhibited the secretion of several cytokines *in vivo* and in splenocytes *in vitro*. We next examined whether PGB could modulate mitogen-induced histologic changes in the spleen.

**Figure 4:**
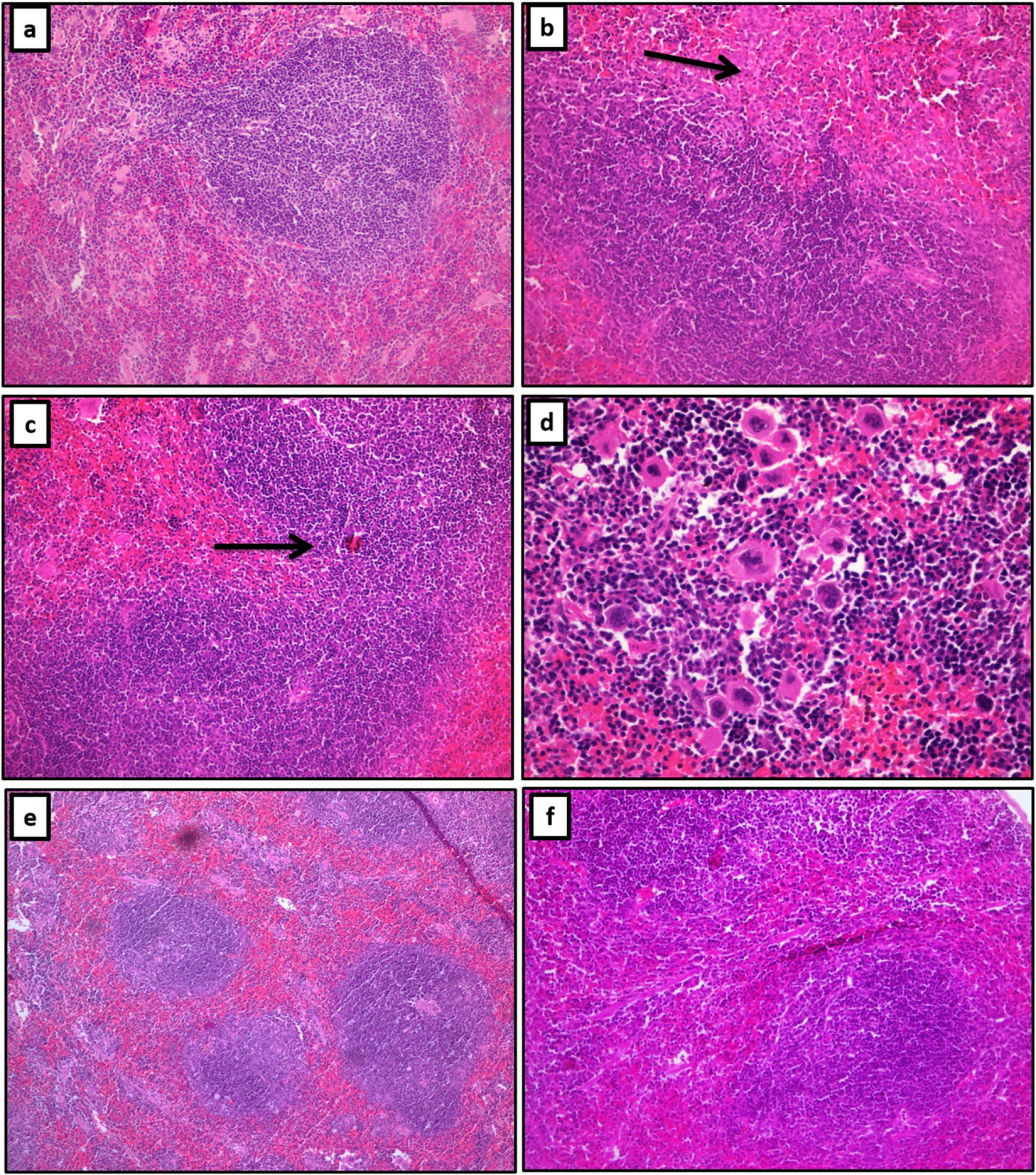
Effect of PGB on ConA- and LPS-induced histologic changes in murine spleen. Mice were either treated with NS only or 16 mg/kg of PGB for 6 days in the presence of 0.1 mg/kg LPS or 10 mg/kg ConA. (**a**) NS only, normal spleen showing a white pulp nodule composed of B lymphocytes, the surrounding red pulp shows a small number of macrophages and megakaryocytes (20X). (**b**) LPS only, an increase in the number of macrophages (arrow) and expansion of the white pulp with fusion of the splenic nodules (20X). (**c**) ConA only, an increase in the white pulp density with fusion of splenic nodules (arrow) (20X). (**d**) ConA only, a noticeable increase in the number of megakaryocytes with loose and compact clustering (40X) (**e**) LPS + PGB, the splenic nodules are small with no fusion and no germinal centre formation. The numbers of macrophages and megakaryocytes are comparable to normal spleen in (**a**) (10X). (**f**) ConA + PGB, the splenic nodules are relatively small with no fusion and no germinal centre formation. The numbers of megakaryocytes and macrophages are comparable to normal spleen in (**a**) (20X). In each group (n = 6). Images were taken using Olympus light microscope.

Representative images of the effect of PGB on spleen histology and on ConA- or LPS-induced inflammatory changes in the spleen are shown in Fig. 4(a–f). Both ConA and LPS induced only moderate changes in the spleens at the doses used in this study (Fig. 4b–d), as compared to the normal untreated spleen (PGB-untreated control group) (Fig. 4a). The white pulp was increased in the form of expanded splenic nodules with germinal centre formation and expanded periarteriolar lymphoid sheaths. In addition, there was an increase in the number of macrophages in the sinuses as well as the number of megakaryocytes that showed compacted clustering. On the other hand, PGB, particularly at 16 mg/kg, ameliorated both ConA- and LPS-induced histologic changes in the spleens, where the spleens of mice treated with 16 mg/kg PGB in the presence of ConA or LPS (Fig. 4e,f) showed comparable number of macrophages and megakaryocytes to those seen in the PGB-untreated control group (Fig. 4a). In addition, the morphology of the white pulp was less prominent with no germinal centre formation and with a reduction in the periarteriolar lymphoid sheath. PGB, at 8 µg/kg, did not exert any significant effects on ConA- or LPS-induced histologic changes in the spleens (data not shown). Furthermore, PGB alone had no effect on spleen histology (data not shown). In conclusion, 16 mg/kg PGB attenuated ConA- and LPS-induced changes in murine spleen. Chronic treatment protocols are required to further investigate these effects.

## Discussion

In this short-term exposure study, we show for the first time that PGB has significant inhibitory effect on LPS/ConA-induced cytokines secretion both *in vivo*, and *in vitro* in splenocytes, and on the basal secretion of some of the cytokines tested. Besides, PGB ameliorated mitogen-induced inflammatory changes in the spleen. The results of this study might give some clues to the effect of PGB on the inflammation observed in PGB-indicated conditions (neuropathic pain conditions, fibromyalgia, epilepsy and generalized anxiety disorders).

Peripheral inflammation is observed in the primary PGB-indicated conditions which all share elevated levels of IL-6, TNF-α, IL-1β and IL-2 cytokines^7–17^. Mitogen (LPS or ConA)-induced models of inflammation has long been used to assess the effects of drugs on macrophages- and T-cells-mediated proinflammatory cytokines production^34^ and were also used to study the inflammation in PGB-indicated conditions^21–24,26^. Therefore, we used these models of inflammation to investigate the effect of PGB on the production of the above-mentioned cytokines. LPS is a potent stimulator of TLR4 in macrophages, resulting in the production of IL-6, TNF-α and IL-1β^33^. Hence, LPS was used in this study to stimulate PMs *in vitro* and to induce these cytokines *in vivo*. ConA is a potent stimulator of T-cells, resulting in enhanced IL-2 secretion, as well as TNF-α and IL-6 secretion^33,35^. As IL-2 is induced after ConA stimulation, but not after LPS stimulation^33,36^, we assessed the effect of PGB on IL-2 secretion in ConA-model only, both *in vivo* and in splenocytes *in vitro*. Thus, employment of both models allowed the investigation of the four cytokines tested. Besides, the investigation on peritoneal macrophages is supported by the emerging role of monocytes in promoting brain inflammation and exacerbation of neuronal damage in neuroinflammatory conditions such as status epilepticus^37^. The doses of the mitogens, and the time points for cytokine measurements were selected based on previous studies and on optimization experiments^21–25,28,30^. In LPS-model of neuroinflammatory conditions, seizure and anxiety, LPS was used at doses of 0.01–3 mg/kg^21–24,28^. In this study, a dose of 0.1 mg/kg was used to induce inflammation in mice while keeping minimal lethality, and to allow the moderate anti-inflammatory effect of PGB to be significantly observed. In the ConA-model of inflammation, the dose and the concentration used were based on our previous studies^28,30^. The 2-hour time point for cytokines measurement after LPS administration *in vivo* was previously shown to significantly increase IL-6, TNF-α, IL-1β and IL-2 levels in LPS-induced inflammation in mice^25,30^. *In vitro*, cytokines were previously measured after 24, 48 and 72 hours of mitogen administration in BALB/c mice^28,30^.

The data presented here show that PGB had no effect on splenocytes proliferation *in vitro* neither in the presence nor in the absence of ConA (Supplementary Table S1). This is consistent with a previous report where PGB had no effect on the proliferation of phytohemagglutinin (a mitogen)-stimulated splenocytes^20^. However, we demonstrated a significant inhibitory effect of PGB on cytokines secretion *in vivo*, and in splenocytes but not in PMs *in vitro*. Whilst our data were similar to previous reports in the inhibition of IL-6, IL-1β and TNF-α secretion^19,38–41^, careful comparisons should be applied as those reports used different mitogens (carrageenan)^19^, different doses of PGB (at least 30 mg/kg of PGB)^19^, different inflammatory models (hyperglycemic stroke^39^ or chronic constriction injury models^40,41^), different sample for the assessment of cytokines levels (brain tissue^39^ or nerve tissue^40,41^) or even different animal species (rat)^19,38–41^. Noteworthy, species, strain- and sex-associated differences in the regulation of some immune responses have been previously reported and similarity in responses is dependent on the outcomes measured^42–45^. Consequently, our study was the only report that investigated the anti-inflammatory effect of PGB in LPS/ConA-models of inflammation in BALB/c, employing comparable doses of PGB to the approved human therapeutic doses, and utilizing the mitogens which were previously used to study PGB-indicated conditions^21–24,26^.

Similar to previous reports^46,47^, we showed undetectable basal IL-1β levels both *in vivo* and *in vitro*. *In vivo*, PGB led to a significant inhibition of LPS-induced IL-1β secretion. However, ConA-induced IL-1β secretion was only slightly inhibited. *In vitro*, IL-1β secretion was only induced in PMs after 72-hour incubation with 100 ng/ml LPS, and was not inhibited by PGB (Supplementary Fig. S1).

We demonstrated herein that PGB had no effect on the proliferation or cytokine secretion in PMs *in vitro* (Supplementary Table S1 and Supplementary Fig. S1). However, using the LPS-induced model of inflammation, other antiepileptic drugs such as lamotrigine significantly inhibited basal and LPS-induced IL-6 and TNF-α secretion in PMs *in vitro*^30^. This can be attributed to the structural differences between the two drugs.

Interestingly, our results showed that PGB not only significantly reduced mitogen-induced cytokines secretion but also the basal secretion. This further supports the anti-inflammatory effect of PGB and might suggest the possible utilization of similar molecular mechanisms for the reduction of both the basal and mitogen-induced cytokines secretion in BALB/mice. The exact mechanism of this anti-inflammatory effect is still not clear. It could be due to a direct effect in modulating the activity of some transcription factors, particularly nuclear factor-kappaB (NF-κB), as reported before^48^. Besides, there is an increasing body of evidence for the expression of voltage-dependent calcium channels in immune cells^49^, thus it might be possible that as PGB alter the function of the voltage-dependent calcium channels in the brain, it might also act on the voltage-dependent calcium channels in the immune cells. However, this theory requires further investigation. Similar basal anti-inflammatory effect was observed before by antiepileptic drugs that inhibits neuronal sodium channels like vinpocetine, carbamazepine and lamotrigine^30,50^. This basal inhibition might be clinically relevant to the current PGB-indicated conditions where elevated basal levels of these cytokines were clinically observed^7–17^.

In both models tested here, and as reported before^33,51^, we showed that LPS/ConA resulted in the elevation of the proinflammatory cytokines IL-6, TNF-α and IL-1β, and that IL-2 was specifically induced with ConA treatment. IL-6 is a pleiotropic cytokine which has multiple effects on immune and non-immune cells, including the nervous system^52^. In the nervous system, IL-6 has a key role in the normal homeostasis of neuronal tissue, while its overexpression in the brain results in neurodegeneration^52^. Both TNF-α and IL-1β are proinflammatory cytokines that induce the inflammatory response, TNF-α additionally regulates the inflammatory response by limiting the extent and the duration of inflammation as demanded^53^. Interestingly, IL-1β and TNF-alpha are inducers for the production of IL-6 through activation of transcription factors^53^. Therefore, PGB inhibitory effect on IL-6 might be a direct effect, or indirect effect through inhibition of IL-1β and/or TNF-α expression. IL-2, among other effects, enhances the proliferation of effector T cells and B cells^53^. Thus, PGB inhibitory effect on IL-2 production might alter the adaptive immune response. The effect of PGB on this complex interaction among the tested proinflammatory cytokines requires further investigation.

The spleen is a lymphoid organ that is composed of a red pulp and a white pulp. The white pulp contains a large reserve of lymphocytes and monocytes^54^. We showed here that both LPS and ConA resulted in expansion of the white pulp and an increase in the number of macrophages in the sinuses as well as the number of megakaryocytes. These effects were all attenuated with 16 µg/kg PGB treatment to almost the levels observed in the PGB-untreated control group (Normal saline). This supports our results of the inhibition of cytokines secretion by isolated splenocytes *in vitro*. Similarly, PGB was reported to ameliorate the inflammatory damage caused by LPS, however, in the hippocampus and cerebellum in rats^18^. Of note, although in our study PGB attenuated mitogen-induced inflammatory changes in the spleen, a tumorigenic effect of PGB was demonstrated before, where chronic administration of 1000–5000 mg/kg of PGB for up to 1 year induced hemangiosarcoma in mice and resulted an increase in the number of macrophages and megakaryocytes in mouse spleen^55^. Therefore, the immunomodulatory effects of PGB seems to be dose and time dependent.

In the current study, PGB demonstrated some degree of differential effects at the different experimental setting employed. In splenocytes, all the cytokines tested were significantly inhibited by PGB treatment with the exception of IL-1β. Similarly, previous reports showed differential inhibitory effects of anti-inflammatory compounds on cytokines secretion in splenocytes^56,57^. Such effects varied based on the cytokine investigated, the mitogen used to stimulate splenocytes, and the concentration of the inhibitors^56,57^. In addition, PGB demonstrated here a cell-based differential effect on cytokine secretion, unlike in splenocytes, PGB did not inhibit cytokine secretion in macrophages at the experimental setting used in our study (Supplementary Fig. S1). This could be attributed to the different receptor expression in the different cell population, and therefore, their responsiveness to PGB. Such differential responsiveness of immune cells to anti-inflammatory agents was observed before^58^. Our data showed that both IL-6 and TNF-α were inhibited by PGB *in vivo*. However, PGB inhibited LPS-induced but not ConA-induced IL-1β secretion, and inhibited ConA-induced but not LPS-induced IL-2 secretion *in vivo*. These *in vivo* responses to PGB resulted from the complex interaction of the immune system elements in the *in vivo* environment, which resulted in the observed mitogen-specific differences in PGB effects. Other antiepileptic agents had also shown differential effects on cytokines secretion, where lamotrigine has been shown to inhibit IL-2, IL-1β and TNF-α secretion, but not IL-6 secretion, in stimulated-human blood *in vitro*^4^.

Of note, in the current report we have focused on the effects of PGB on peripheral inflammatory responses. Although neuroinflammation *per se* was not investigated in this study, our results might give some clues to the effect of PGB on peripheral inflammation observed in PGB-indicated conditions (neuropathic pain conditions, fibromyalgia, epilepsy and generalized anxiety disorders) where inflammatory mechanisms and elevation of proinflammatory cytokines, such as IL-6, TNF-α, IL-1β and IL-2 have been documented^7–11,16,17^. Besides, compared to our study, similar model of inflammation and comparable doses of LPS were employed before to study these conditions^21–24^. Additionally, it was previously shown that LPS-induced systemic inflammation lead to neuroinflammation in mice^25^. Importantly, targeting IL-6, TNF-α and IL-1β proinflammatory cytokines has been previously proposed for the management of neuropathic pain conditions and epilepsy^8,59,60^. Therefore, the therapeutic benefit of PGB in the management of these conditions might involve the anti-inflammatory mechanisms demonstrated in this study, in combination with the mechanism involving binding to the voltage-dependent calcium channels in the brain^6^. However, careful interpretation of the data in clinical setting is required as this study represents a short-term exposure protocol while PGB is clinically used for long-term treatment. Development of chronic exposure protocols and chronic models of inflammation is demanded to further clarify the long-term anti-inflammatory effects of PGB. Besides, the effects of PGB on inflammatory responses within the CNS could be investigated in future studies using CSF samples or microglial cell cultures.

## Conclusion

The data presented in the current study demonstrated for the first time a significant peripheral anti-inflammatory effect of PGB on murine LPS/ConA-induced cytokines secretion both *in vivo*, and *in vitro* in splenocytes. Besides, PGB attenuated mitogen-induced inflammatory changes in murine spleen. PGB, however, had no effect on cytokine secretion in PMs *in vitro*. Our findings may extend the current state of knowledge regarding the role of PGB in modulating the immune system in neuroinflammatory conditions such as neuropathic pain conditions, fibromyalgia, epilepsy and generalized anxiety disorders. This anti-inflammatory effect might be adjunctive to the documented mechanism of action of PGB (binding to the voltage-dependent calcium channels in the brain) in treating these conditions. Further studies, however, are necessary to investigate the effects of PGB on cytokine levels within the CNS and to investigate these effects during chronic exposure protocols.

## Supplementary information

Supplementary information.

## Data Availability

The dataset analyzed during the current study is available from the corresponding author on reasonable request.

## References

[1] Godhwani N, Bahna SL (2016). Antiepilepsy drugs and the immune system. Ann. Allergy Asthma Immunol..

[2] Blaszczyk B, Lason W, Czuczwar SJ (2015). Antiepileptic drugs and adverse skin reactions: an update. Pharmacol. Rep..

[3] Himmerich H (2014). Modulation of cytokine production by drugs with antiepileptic or mood stabilizer properties in anti-CD3- and anti-Cd40-stimulated blood *in vitro*. Oxid. Med. Cell Longev..

[4] Himmerich H (2013). Impact of mood stabilizers and antiepileptic drugs on cytokine production *in-vitro*. J. Psychiatr. Res..

[5] Maes M, Bosmans E, Calabrese J, Smith R, Meltzer HY (1995). Interleukin-2 and interleukin-6 in schizophrenia and mania: effects of neuroleptics and mood stabilizers. J. Psychiatr. Res..

[6] Houghton KT (2017). Biological rationale and potential clinical use of gabapentin and pregabalin in bipolar disorder, insomnia and anxiety: protocol for a systematic review and meta-analysis. BMJ Open..

[7] Sommer C, Leinders M, Uceyler N (2018). Inflammation in the pathophysiology of neuropathic pain. Pain..

[8] Hung AL, Lim M, Doshi TL (2017). Targeting cytokines for treatment of neuropathic pain. Scand. J. Pain..

[9] Iannuccelli C (2010). Pathophysiology of fibromyalgia: a comparison with the tension-type headache, a localized pain syndrome. Ann. N. Y. Acad. Sci..

[10] Ernberg M (2018). Plasma Cytokine Levels in Fibromyalgia and Their Response to 15 Weeks of Progressive Resistance Exercise or Relaxation Therapy. Mediators Inflamm..

[11] Backryd E, Tanum L, Lind AL, Larsson A, Gordh T (2017). Evidence of both systemic inflammation and neuroinflammation in fibromyalgia patients, as assessed by a multiplex protein panel applied to the cerebrospinal fluid and to plasma. J. Pain. Res..

[12] Alapirtti T (2018). The production of IL-6 in acute epileptic seizure: a video-EEG study. J. Neuroimmunol..

[13] De Vries EE (2016). Inflammatory mediators in human epilepsy: a systematic review and meta-analysis. Neurosci. Biobehav. Rev..

[14] Jarvela JT, Lopez-Picon FR, Plysjuk A, Ruohonen S, Holopainen IE (2011). Temporal profiles of age-dependent changes in cytokine mRNA expression and glial cell activation after status epilepticus in postnatal rat hippocampus. J. Neuroinflamm.

[15] Pacifici R (1995). Cytokine production in blood mononuclear cells from epileptic patients. Epilepsia.

[16] De Sarro G, Rotiroti D, Audino M, Gratteri S, Nisticó G (1994). Effect of interleukin-2 on various models of experimental epilepsy in DBA/2 mice. Neuroimmunomodulation.

[17] Costello H, Gould RL, Abrol E, Howard R (2019). Systematic review and meta-analysis of the association between peripheral inflammatory cytokines and generalised anxiety disorder. BMJ Open..

[18] Aslankoc R, Savran M, Ozmen O, Asci S (2018). Hippocampus and cerebellum damage in sepsis induced by lipopolysaccharide in aged rats - pregabalin can prevent damage. Biomed. Pharmacother..

[19] Kilic FS (2018). Pregabalin attenuates carrageenan-induced acute inflammation in rats by inhibiting proinflammatory cytokine levels. Eurasian J. Med..

[20] Jang Y, Song HK, Yeom MY, Jeong DC (2012). The immunomodulatory effect of pregabalin on spleen cells in neuropathic mice. Anesth. Analg..

[21] Zhao J (2019). Neuroinflammation induced by lipopolysaccharide causes cognitive impairment in mice. Sci. Rep..

[22] Barton SM (2019). Lipopolysaccharide Induced Opening of the Blood Brain Barrier on Aging 5XFAD Mouse Model. J. Alzheimers Dis..

[23] Eun BL, Abraham J, Mlsna L, Kim MJ, Koh S (2015). Lipopolysaccharide potentiates hyperthermia-induced seizures. Brain Behav..

[24] Sulakhiya K (2016). Lipopolysaccharide induced anxiety- and depressive-like behaviour in mice are prevented by chronic pre-treatment of esculetin. Neurosci. Lett..

[25] Biesmans S (2013). Systemic immune activation leads to neuroinflammation and sickness behavior in mice. Mediators Inflamm..

[26] Shanklin DR, Stevens MV, Hall MF, Smalley DL (2000). Environmental immunogens and T-cell- mediated responses in fibromyalgia: evidence for immune dysregulation and determinants of granuloma formation. Exp. Mol. Pathol..

[27] Elhayek SY, Fararjeh MA, Assaf AM, Abu-Rish EY, Bustanji Y (2018). Immunomodulatory effects of tigecycline in Balb/c mice. Acta Pharm..

[28] Abu-Rish EY, Elhayek SY, Mohamed YS, Hamad I, Bustanji Y (2017). Evaluation of immunomodulatory effects of lamotrigine in BALB/c mice. Acta Pharm..

[29] Fararjeh M, Mohammad MK, Bustanji Y, Alkhatib H, Abdalla S (2008). Evaluation of immunosuppression induced by metronidazole in Balb/c mice and human peripheral blood lymphocytes. Int. Immunopharmacol..

[30] Abu-rish EY, Dahabiyeh LA, Bustanji Y, Mohamed YS, Browning MJ (2018). Effect of lamotrigine on *in vivo* and *in vitro* cytokine secretion in murine model of inflammation. J. Neuroimmunol..

[31] Berry D, Millington C (2005). Analysis of pregabalin at therapeutic concentrations in human plasma/serum by reversed-phase HPLC. Ther. Drug. Monit..

[32] Abu-rish EY (2016). Evaluation of antiproliferative activity of some traditional anticancer herbal remedies from Jordan. Trop. J. Pharm. Res..

[33] Bellanti, J. A. Immunology IV: *Clinical Applications in Health and Disease* (ed. 3) I Care Press, Bethesda (MD), pp 287–363. (2012).

[34] Tryphonas H (2001). Approaches to detecting immunotoxic effects of environmental contaminants in humans. Environ. Health Perspect..

[35] Dwyer JM, Johnson C (1981). The use of concanavalin A to study the immunoregulation of human T cells. Clin. Exp. Immunol..

[36] Rossol M (2011). LPS-induced cytokine production in human monocytes and macrophages. Crit. Rev. Immunol..

[37] Varvel NH (2016). Infiltrating monocytes promote brain inflammation and exacerbate neuronal damage after status epilepticus. Proc. Natl. Acad. Sci. USA.

[38] Ozmen Ozlem, Topsakal Senay (2019). Pregabalin Ameliorates Lipopolysaccharide-Induced Pancreatic Inflammation in Aged Rats. Endocrine, Metabolic & Immune Disorders - Drug Targets.

[39] Song Y (2017). Effect of pregabalin administration upon reperfusion in a rat model of hyperglycemic stroke: Mechanistic insights associated with high-mobility group box 1. PLoS One.

[40] Khan J, Noboru N, Imamura Y, Eliav E (2018). Effect of Pregabalin and Diclofenac on tactile allodynia, mechanical hyperalgesia and pro inflammatory cytokine levels (IL-6, IL-1β) induced by chronic constriction injury of the infraorbital nerve in rats. Cytokine.

[41] Khan J, Alghamdi H, Anwer MM, Eliav E, Ziccardi V (2016). Role of Collagen Conduit With Duloxetine and/or Pregabalin in the Management of Partial Peripheral Nerve Injury. J. Oral. Maxillofac. Surg..

[42] Garcia-Pelayo MC, Bachy VS, Kaveh DA, Hogarth PJ (2015). BALB/c mice display more enhanced BCG vaccine induced Th1 and Th17 response than C57BL/6 mice but have equivalent protection. Tuberculosis..

[43] Weinstein Y, Ran S, Segal S (1984). Sex-associated differences in the regulation of immune responses controlled by the MHC of the mouse. J. Immunol..

[44] Kay E, Gomez-Garcia L, Woodfin A, Scotland RS, Whiteford JR (2015). Sexual dimorphisms in leukocyte trafficking in a mouse peritonitis model. J. Leukoc. Biol..

[45] Lam D, Lively S, Schlichter LC (2017). Responses of rat and mouse primary microglia to pro- and anti-inflammatory stimuli: molecular profiles, K^+^ channels and migration. J. Neuroinflammation..

[46] Lee SB (2018). Kaempferol 7-O-β-D-glucoside isolated from the leaves of cudrania tricuspidata inhibits LPS-induced expression of pro-inflammatory mediators through inactivation of NF-κB, AP-1, and JAK-STAT in RAW 264.7 macrophages. Chem. Biol. Interact..

[47] Zhao F (2018). Titanium dioxide nanoparticle stimulating pro-inflammatory responses *in vitro* and *in vivo* for inhibited cancer metastasis. Life Sci..

[48] Beghi E, Shorvon S (2011). Antiepileptic drugs and the immune system. Epilepsia.

[49] Davenport B, Li Y, Heizer JW, Schmitz C, Perraud AL (2015). Signature Channels of Excitability no More: L-Type Channels in Immune Cells. Front. Immunol..

[50] Gómez CD, Buijs RM, Sitges M (2014). The anti-seizure drugs vinpocetine and carbamazepine, but not valproic acid, reduce inflammatory IL-1β and TNF-α expression in rat hippocampus. J. Neurochem..

[51] Zheng W (2018). Lepidium meyenii Walp Exhibits Anti-Inflammatory Activity against ConA-Induced Acute Hepatitis. Mediators Inflamm..

[52] Rothaug M, Becker-Pauly C, Rose-John S (2016). The role of interleukin-6 signaling in nervous tissue. Biochim. Biophys. Acta..

[53] Akdis M (2016). Interleukins (from IL-1 to IL-38), interferons, transforming growth factor β, and TNF-α: Receptors, functions, and roles in diseases. J. Allergy Clin. Immunol..

[54] Cesta MF (2006). Normal structure, function, and histology of the spleen. Toxicol. Pathol..

[55] Criswell KA (2012). Key components of the mode of action for hemangiosarcoma induction in pregabalin-treated mice: evidence of increased bicarbonate, dysregulated erythropoiesis, macrophage activation, and increased angiogenic growth factors in mice but not in rats. Toxicol. Sci..

[56] Wang X (2017). Inhibition of cytokine response to TLR stimulation and alleviation of collagen-induced arthritis in mice by Schistosoma japonicum peptide SJMHE1. J. Cell. Mol. Med..

[57] Lin WC, Lin JY (2011). Five bitter compounds display different anti-inflammatory effects through modulating cytokine secretion using mouse primary splenocytes *in vitro*. J. Agric. Food Chem..

[58] Cho JY (2007). Immunomodulatory effect of nonsteroidal anti-inflammatory drugs (NSAIDs) at the clinically available doses. Arch. Pharm. Res..

[59] Vezzani A, Viviani B (2015). Neuromodulatory properties of inflammatory cytokines and their impact on neuronal excitability. Neuropharmacol..

[60] Vezzani A (2010). ICE/caspase 1 inhibitors and IL-1beta receptor antagonists as potential therapeutics in epilepsy. Curr. Opin. Investig. Drugs.

