# Supplementary material for: Pregabalin inhibits in vivo and in vitro cytokine secretion and attenuates spleen inflammation in Lipopolysaccharide/Concanavalin A -induced murine models of inflammation
Source: Sci Rep. 2020 Mar 4;10:4007. doi: 10.1038/s41598-020-61006-1 (PMC7055236; doi:10.1038/s41598-020-61006-1)
Supplement: Supplementary file 1 — Supplementary information. [file 41598_2020_61006_MOESM1_ESM.pdf]

# Pregabalin inhibits *in vivo* and *in vitro* cytokine secretion and attenuates spleen inflammation in Lipopolysaccharide/Concanavalin A -induced murine models of inflammation

Eman Y. Abu-rish[1,*], Ahmad T. Mansour[2], Hebah T. Mansour[1], Lina A. Dahabiyeh[3], Shereen M. Aleidi[1], Yasser Bustanji[1,4]

[1] Department of Biopharmaceutics and Clinical Pharmacy, School of Pharmacy, The University of Jordan, Amman, 11942, Jordan.

[2] Department of Pathology and Microbiology and Forensic Medicine, School of Medicine, The University of Jordan, Amman, 11942, Jordan.

[3] Department of Pharmaceutical Sciences, School of Pharmacy, The University of Jordan, Amman, 11942, Jordan.

[4] Hamdi Mango Centre for Scientific Research, The University of Jordan, Amman, Jordan

*__Correspondence__: E.Y. Abu-rish, Department of Biopharmaceutics and Clinical Pharmacy, School of Pharmacy, The University of Jordan, Amman, PO Box 11942, Jordan.

Email: e.aburish@ju.edu.jo (E.Y. Abu-rish)

| | | Without Mitogen (Cell viability, OD ± SEM) | | | With Mitogen (Cell viability, OD ± SEM) | | |
|---|---|---|---|---|---|---|---|
| | **PGB (µg/ml)** | **24 hours** | **48 hours** | **72 hours** | **24 hours** | **48 hours** | **72 hours** |
| **Splenocytes** | **0** | 0.126± 0.0034 | 0.133± 0.0044 | 0.124± 0.0048 | 0.240± 0.0188 | 0.394± 0.0147 | 0.597± 0.0337 |
| | **1.5** | 0.142± 0.0067 | 0.126± 0.0040 | 0.120± 0.0091 | 0.213± 0.0107 | 0.387± 0.0122 | 0.595± 0.0394 |
| | **3** | 0.131± 0.0100 | 0.129± 0.0300 | 0.152± 0.0095 | 0.235± 0.0134 | 0.364± 0.0134 | 0.562± 0.0214 |
| | **10** | 0.114± 0.0032 | 0.106± 0.0149 | 0.149± 0.0156 | 0.230± 0.0115 | 0.371± 0.0047 | 0.587± 0.0396 |
| | **30** | 0.142± 0.0040 | 0.134± 0.0127 | 0.147± 0.0067 | 0.244± 0.0142 | 0.401± 0.0074 | 0.608± 0.0136 |
| | **60** | 0.135± 0.0085 | 0.111± 0.0134 | 0.134± 0.0071 | 0.249± 0.0082 | 0.402± 0.0267 | 0.606± 0.0453 |
| ***P*-value** | | 0.0763 | 0.3721 | 0.1444 | 0.4946 | 0.3976 | 0.9363 |
| **PMs** | **0** | 0.328± 0.0333 | 0.360± 0.0077 | 0.466± 0.0273 | 0.312± 0.0154 | 0.473± 0.0104 | 0.468± 0.0363 |
| | **1.5** | 0.387± 0.0244 | 0.430± 0.0225 | 0.467± 0.0069 | 0.306± 0.0066 | 0.459± 0.0037 | 0.422± 0.0318 |
| | **3** | 0.411± 0.0339 | 0.406± 0.0146 | 0.465± 0.0184 | 0.333± 0.0067 | 0.505± 0.0229 | 0.445± 0.0414 |
| | **10** | 0.426± 0.0149 | 0.414± 0.0185 | 0.407± 0.012 | 0.331± 0.0118 | 0.469± 0.0170 | 0.433± 0.0142 |
| | **30** | 0.417± 0.0144 | 0.414± 0.0077 | 0.418± 0.0613 | 0.331± 0.0131 | 0.447± 0.0079 | 0.495± 0.0200 |
| | **60** | 0.410± 0.0278 | 0.392± 0.0296 | 0.399± 0.0096 | 0.273± 0.0251 | 0.448± 0.0135 | 0.487± 0.0071 |
| ***P*-value** | | 0.1412 | 0.2132 | 0.3857 | 0.0822 | 0.1077 | 0.3926 |

**Supplementary Table S1.** Effect of PGB on splenocytes and PMs cell viability in the presence or absence of ConA or LPS, respectively. 1 µg/ml of ConA were used in splenocytes cultures and 100 ng/ml of LPS were used in PMs cultures. ConA**:** concanavalin A, PMs: peritoneal macrophages, LPS: lipopolysaccharides, OD: optical density, SEM: standard error of the mean. Data represent means ± SEM, n = 3 (for each experiment 4 mice were used for pooled splenocytes culture preparation, while 12 mice were used to prepare pooled peritoneal macrophages cultures), ANOVA, The *P*-value refers to the comparison of the effect of PGB at the different concentrations tested (0-60 µg/ml) at each time point (24, 48 and 72 hours).

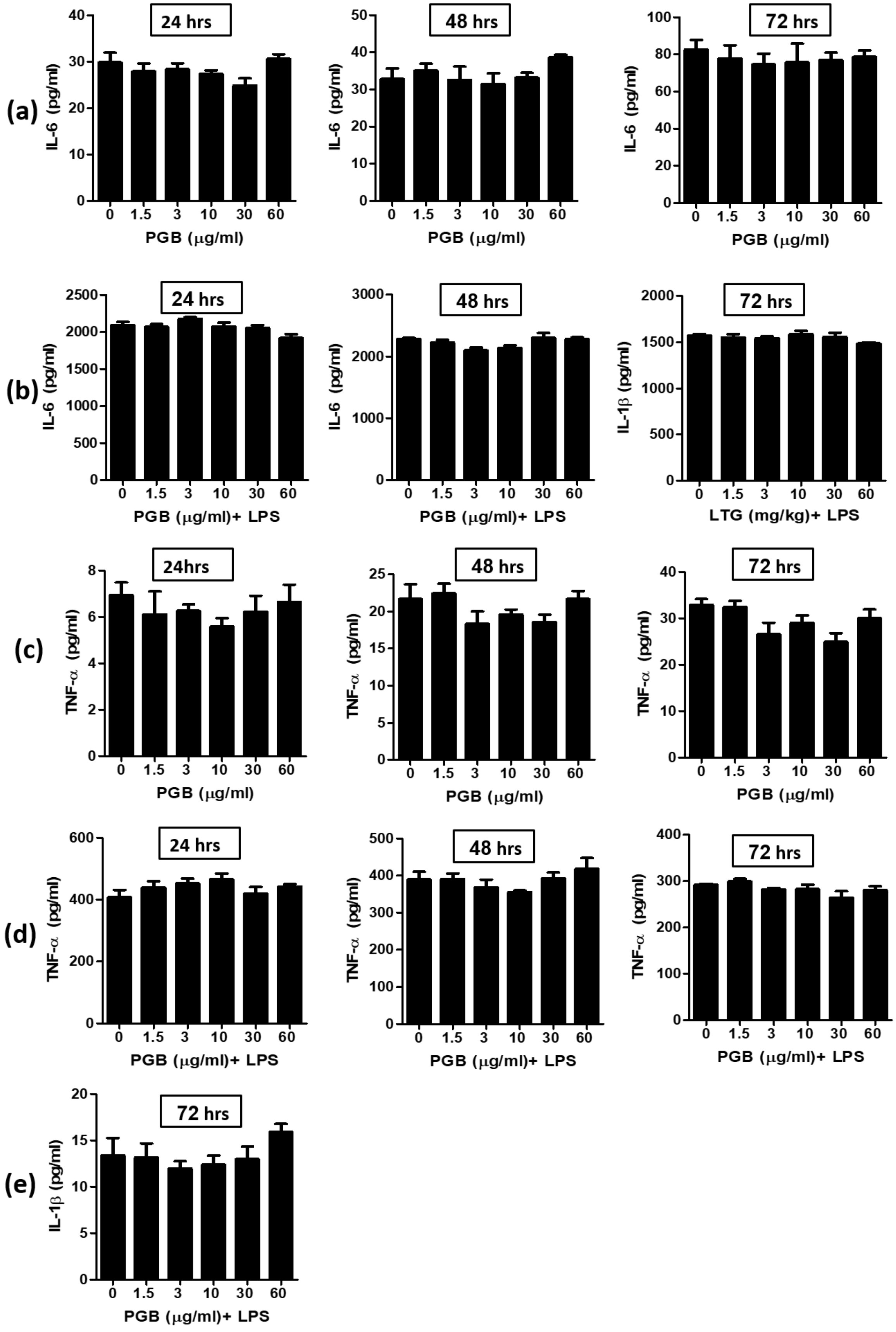
(a)
24 hrs
IL-6 (pg/ml)
PGB (µg/ml)
48 hrs
IL-6 (pg/ml)
PGB (µg/ml)
72 hrs
IL-6 (pg/ml)
PGB (µg/ml)
(b)
24 hrs
IL-6 (pg/ml)
PGB (µg/ml)+ LPS
48 hrs
IL-6 (pg/ml)
PGB (µg/ml)+ LPS
72 hrs
IL-1β (pg/ml)
LTG (mg/kg)+ LPS
(c)
24hrs
TNF-α (pg/ml)
PGB (µg/ml)
48 hrs
TNF-α (pg/ml)
PGB (µg/ml)
72 hrs
TNF-α (pg/ml)
PGB (µg/ml)
(d)
24 hrs
TNF-α (pg/ml)
PGB (µg/ml)+ LPS
48 hrs
TNF-α (pg/ml)
PGB (µg/ml)+ LPS
72 hrs
TNF-α (pg/ml)
PGB (µg/ml)+ LPS
(e)
72 hrs
IL-1β (pg/ml)
PGB (µg/ml)+ LPS

**Supplementary Fig. S1.** Effect of PGB on IL-6, TNF-α and IL-1β secretion in PMs. (a) basal IL-6 secretion, (b) LPS-induced IL-6 secretion, (c) basal TNF-α secretion, (d) LPS-induced TNF-α secretion, (e) LPS-induced IL-1β secretion. PMs were treated with PGB alone (1.5, 3, 10, 30, 60 μg/ml) or were pre-treated with PGB for 1 hour and then co-treated with LPS (100 ng/ml). Cells were incubated for 24, 48 and 72 hours. LPS: lipopolysaccharide, PGB, Pregabalin, (Data represent mean ± SEM; n = 3; ANOVA; Dunnett's post-hoc analysis)
